# The Effect of Emotional Dissonance and Mental Load on Need for Recovery and Work Engagement among Italian Fixed-Term Researchers

**DOI:** 10.3390/ijerph18010099

**Published:** 2020-12-25

**Authors:** Francesco Pace, Giulia Sciotto

**Affiliations:** 1 Department of Economics, Business and Statistics, University of Palermo, 90128 Palermo, Italy; 2 Department of Psychology, Educational Sciences and Human Movement, University of Palermo, 90128 Palermo, Italy; giulia.sciotto@unipa.it

**Keywords:** work stress factors, job resources, university, emotional dissonance, mental load, need for recovery, work engagement, job insecurity

## Abstract

Although many studies have been conducted to evaluate the risk and protective factors on psychological health among academic staff, little attention has been paid to fixed-term researchers, the weakest figures in the academic context. By using the Job Demands–Resources model as theoretical framework, we investigated: (1) the role of some job demands (workload, mental load, and emotional dissonance) in predicting the need for recovery; (2) the role of some job resources (independence, career opportunities, and work–life balance) in predicting work engagement; and (3) the moderating role of the contract type (more or less precarious). We focused in particular on emotional dissonance (the discrepancy between emotions that need to be displayed and what is really felt), assuming its unique role in predicting fatigue. Results of structural equation modeling analysis generally supported our hypotheses and highlighted a so far undiscovered path between mental load and work engagement. Specifically, mental load leads to fatigue only indirectly through workload and emotional dissonance, while significantly predicting the absorption and the dedication of fixed-term Italian researchers. The latter relationship was also moderated by the contract type, so that mental load predicts dedication especially among researchers in the most precarious condition.

## 1. Introduction

The role of university researchers in Italy has undergone profound changes in recent years, although it remains a central element of the university recruitment process, given its function of ferrying candidates to superior and permanent positions. To better understand the objectives of this paper, some clarifications are needed. In Italy, before the reform implemented in 2010 (Law 240/10), researchers had permanent contracts. With the 2010 reform, a fundamental change of contractual framework is introduced: the figure of the permanent researcher is depleted and definitively replaced by the fixed-term researcher (henceforth called “RTD”, acronym from the Italian “Ricercatore a Tempo Determinato”). For the first time in Italy, the first step of the university career has now been made precarious, amid many protests. Within this renewed category there are two types of contract, only one of which provides for a facilitated access procedure to the role of associate professor. They are “type a” RTDs and “type b” RTDs, henceforth referred to as “RTDa” and “RTDb” respectively. They are very similar in terms of institutional tasks and duties (both provide research and teaching activities, with no difference between type a and type b in terms of overall workload) and profoundly different in terms of career possibilities. Only RTDb have the opportunity, once the contract is completed and after obtaining the National Scientific Qualification, to reach the position of associate professors; RTDa, on the other hand, have no other possibility of career advancement than to access a type b contract and begin a new precarious path. Type a researchers’ career opportunities, in terms of continuity of employment, depend essentially on the availability of local funds, while the only obstacle to the future within the university of the type b researchers, once they become RTDb, is passing the National Scientific Qualification, which depends primarily on their ability to place their research work in the editorial context. 

Many studies have been conducted to evaluate the risk and protective factors among academic staff [1,2,3,4,5,6,7,8]; however, little attention has been paid to fixed-term researchers, the weakest figures in the academic context. Furthermore, while academic workload has been extensively investigated, little attention has been given to both mental load and emotional dissonance as job stressors. The latter has been studied several times in the context of teaching, but only in school teaching, rarely in university, and especially never among fixed-term researchers. Usually, when the fixed-term categories are examined, the focus is on the interference of job insecurity as moderator in the relationships that lead stressors or resources to develop work-related stress or engagement. Indeed, job precarity has been profusely linked to negative job-related attitudes among university staff and defined it as a threat to academics’ resources [4]. However, it is our opinion that focusing on the aspect of precariousness, when RTDs’ work-related stress is examined, might not be enough. The career advancements of university researchers are subject to the carrying out and subsequent publication of their research projects. Therefore, research tasks have an objectively greater importance than didactic tasks, which may often be rather uncomfortable or unpleasant due to the pressure of the “publish or perish” philosophy or, more in general, to the unbalance between the high commitment requested to teach and the low attention given to career development. As a consequence, teaching often brings with itself a certain contradiction between teachers’ true feelings and the expected emotions that are supposed to be displayed. This discrepancy between faked and actually experienced emotion is called emotional dissonance and is an aspect of RTDs’ job that cannot be ignored, since it is believed to be a source of strain that severely threatens well-being [9,10,11].

The aim of this study is to focus on a category of temporary workers largely overlooked by the recent literature on work-related stress in academic contexts: fixed-term researchers. We investigated the role of some academic job demands (workload, mental load and emotional dissonance) in predicting a greater need to recover physical and mental energies at the end of the working day. We focused in particular on emotional dissonance, as a particularly influential stress factor in social work [1]. We hypothesized that, being a sample of workers still at the beginning of their academic career, more experienced and versed in research activities than in teaching and in close contact with students, having to pretend emotions that are not actually felt could worsen their levels of well-being, increasing their need for recovery more than any other job demands. At the same time, we also investigated positive factors, such as the degree of independence in carrying out one’s work, the career opportunities offered by the organization and the work–life balance. We assumed that these would have a positive influence on work engagement, while decreasing the stress outcome levels. A comprehensive theoretical framework, the Job Demands–Resources model (JD-R model) [12,13], was used and applied to the investigation of the relationships between above mentioned organizational stressors and resources, and their combined effects on, respectively, need for recovery and work engagement among fixed-term researchers of Italian universities. In this study, we use the type of contract as an indicator of job insecurity (assuming that type a RTDs have a higher level of job insecurity than type b RTDs) to verify its moderating role in the relationship between job resources and work engagement.

### 1.1. Job Demands–Resources Model

Stress research differentiates between stressors and resources, and assumes that stressors are related to strain variables, whereas resources are assumed to be related to motivational factors and to function as a buffer against stress. To conceptualize how job demands and job resources combine together we decided to use the Job Demands–Resources model [13] as the theoretical framework in which to place the analysis of the mechanisms through which job and personal characteristics lead, on one side, to an affective-motivational state such as work engagement, and on the other side to one of the most common work-related stress symptoms, the need for recovery.

The Job Demands–Resources model [13] proposes that regardless of job type, job characteristics can be distinguished in job demands and job resources. Job demands refer to those physical, psychological, social or organizational aspects of the job that require physical and/or psychological effort and therefore are associated with certain physiological costs. Job resources refer to those aspects that function to reduce job demands, enable achievement of work goals and stimulate personal growth, learning, and development. An important assumption of the model is that the two sets of factors may evoke, respectively, health-impairment processes and motivational processes [14]. According to the motivational processes, job resources may lead to positive organizational outcomes through work engagement. According to the health-impairment process, high job demands (for example, work overload or emotional demands) exhaust employees’ mental and physical resources and may therefore lead to the depletion of energy (*id est*, exhaustion), psychological strain and poor mental health [14,15]. A persistent condition of emotional exhaustion (i.e., burnout) may give rise to adverse individual and work-related outcomes [16]. For instance, research on educational settings revealed that emotional exhaustion decreases the level of job satisfaction and commitment among teachers and, at the same time, it may induce them to quit the teaching profession [17,18]. Job demands and resources also have joint effects [14]. For example, job resources may buffer the impact of job demands in predicting employee health [19] and job demands may boost the impact of job resources on motivation, such that job resources particularly influence motivation when job demands are high [14,20]. This happens because high job demands challenge the individuals to use their personal resources, which are important in alleviating the effects of job demands on stress. Employees are more than just passive receivers of external influences and demands [21]. In fact, individuals with a greater pool of resources are less susceptible to resource loss, while those individuals who do not have access to strong resource pools in the first place are more likely to experience a further loss of resources (“loss spiral”) [14,22]. Ultimately, job demands become stressors only when they exceed employees’ resources, so that they fail to recover from the efforts spent to execute them.

### 1.2. Job Demands and Resources among Academics

As mentioned above, in recent years Italian university researchers have been affected by the transition from permanent contracts to fixed-term contracts. Their workload and mental load, however, have certainly not diminished. Embedded in a performance-based career advancement system, Italian fixed-term researchers are required to constantly develop their research skills, compete to publish peer-reviewed articles in international journals and undertake more entrepreneurial activities [7]. In the meantime, they are also required to do more paperwork and increase their teaching hours in order to respond to the increase in students’ enrollments [8]. Having found a lack of research that takes this peculiar working situation into consideration, in this paper we wanted to highlight the dynamics that lead to fatigue or engagement among the weakest figures in academy. 

Generally, several key factors are commonly associated with stress among academics. These include work overload, time pressure, emotional demands, job insecurity, lack of promotion opportunities, inadequate recognition, inadequate salary, inadequate resources and funding, excessive administrative work, role ambiguity, pressure to attract external funds, poor management, and student interaction [1,3,4,5]. In all of these studies, academic job stressors are linked to professional and personal consequences, such as poor job performance, poor work relationships, low commitment, physical and psychological health problems, and strained personal relationships. 

*Academic workload*. The impact of several factors on academics’ occupational stress and well-being has been investigated [1]. Results showed that demands of work pressure and workload have the most negative impact on academics’ well-being. Workload, the sense of having too much work to do in the time available, has been profusely linked to psychological stress and worsened job performance, which in the academic world is measured in terms of teaching and research work [6,7,23]. Increasing workloads and work-related stress results in less academic time spent on research, publishing, and professional development, decreasing teaching and research standards, and increasing interpersonal conflict in academic staff relationships. The increase in workload has also adversely affected the balance between work–life and personal life, with academics reporting that they spend most of their weekends dealing with their work [4]. 

*Mental load.* A very specific form of academic workload is mental load, the degree of attention and concentration required by the job [24]. Under normal conditions, employees have enough time to recover and recharge their energy after work, in order to perform well the next day. However, in case of insufficient recovery, employees must make additional, compensatory effort in order to successfully complete their work tasks [25]. Under conditions of fatigue, it takes more effort to concentrate or to divide attention between various task elements [20]. Therefore, if the subject is already overworked, this could trigger a vicious circle such that higher workloads correspond to more mental load and, at the same time, less concentration capacity, which increases the need for recovery at the end of the working day. If the recovery fails, the day after the cycle restarts. If high levels of mental workload cumulate and recovery continues to fail, health problems such as chronic stress, depression, or burnout can occur [26]. Assumed that high workload results in stress responses, such as psychosomatic and psychological complaints, it seems, however, that the relationship between mental load and stress is more complicated than that. In fact, even under unfavorable conditions it is possible to work intensively and to be highly activated without feelings of strain or psychosomatic complaints [24]. It may depend on individual factors. For example, individuals with low emotional stability may view a high workload or a complex work assignment as threatening, whereas individuals with high emotional stability may view the same job demands as challenges [14]. These tend to be appraised as stressful demands that have the potential to promote mastery, personal growth, or future gains. As a consequence, employees may perceive them as opportunities to learn, achieve, and demonstrate the type of competence that tends to get rewarded [27]. In virtue of that, challenging demands may not lead to stress outcomes. Mental workload is a dominant element in most jobs; however, there is very little literature that studies its effect on academics’ stress. Therefore, the role that the mental load will have in the sample of researchers (i.e., stressor or challenge factor) is uncertain.

*Emotional dissonance.* All jobs that involve intense interaction with others, including teaching, have the potential to negatively impact on employee well-being, as a consequence of prolonged emotional involvement in the workplace [28]. University professors, for example, are not only required to work on tasks and spend mental and physical effort, but they are also required to manage their emotions as a part of their job. The process of regulating feelings and expressions as part of the work role is called emotional labor [9,11]. Employees perform emotional labor when they regulate their emotional display in an attempt to meet organizationally based expectations specific to their roles [29]. A part or a negative consequence of the emotional labor construct is the so-called emotional dissonance, a form of person-role conflict that occurs when an employee’s expressed emotions are in conformity with organizational norms but do not represent his or her true feelings [10]. Emotional labor can be performed through two strategies: surface acting and deep acting [9]. In surface acting, employees simply alter their displayed feelings, while the inner feelings remain unchanged. Deep acting, however, is the process of controlling internal thoughts and feelings to meet the mandated display rules; it works on modifying arousal or cognitions [30]. The persistent, structural discrepancy between emotions that need to be displayed and what is really felt can produce alienation from one’s own authentic emotions and, in the long term, psychological strain [9,10,31]. Emotional dissonance is indeed considered to be a job demand that predicts work-related stress when combined with other organizational and social variables, especially work overload, time pressure and role conflicts [32]. However, a unique contribution of emotional dissonance was confirmed, as it acts as a particularly relevant stressor for workers in the human services, predicting stress independently of other organizational stressors [33]. In line with these findings, emotional dissonance might be an unsustainable job stressor for academics as well. Faculty members’ job often requires frequent changes of displayed emotions: positive emotions to build enthusiasm, negative emotions to support discipline, and neutrality of emotions to demonstrate fairness and professionalism [10]. Teachers report frequently faking their enthusiasm, because they believe that it is necessary to behave enthusiastically, regardless of their affective state, to increase students’ interest and motivation [31]. While displaying positive expressions, however, any simultaneously experienced negative emotion remains intact, even if masked. In the long run, similarly to other types of self-control, emotional control depletes mental resources and can therefore lead to exhaustion. 

*Job resources*. If this is what stresses academics the most, what motivates them? Previous research suggests that academics are intrinsically motivated by their disciplines and related teaching and research tasks, but extrinsically demotivated by work context factors such as insufficient funding and resources, and poor management practices [1,2,4]. Academics are more likely to express emotional commitment to their universities when roles are clear, achievable, and not overloaded, when job tasks are challenging, and job autonomy, social support, recognition and work–life balance are granted [1,2]. Autonomy on work content and flexibility in daily schedules reduces the likelihood of work-family conflicts and has beneficial effects on stress levels [34], decreasing job strain and providing compensatory resources that have a positive impact on job satisfaction and well-being [35]. Another motivational and engagement boost is offered by the career opportunities [1]. However, although there is already much scientific evidence confirming the positive role of independence and work–life balance in preventing fatigue, when considering career opportunities, the discussion enters a purely exploratory context, since there are no studies investigating this aspect among academic fixed-term researchers.

### 1.3. Need for Recovery and Work Engagement

According to the Job Demands–Resources model [14], the combination of job demands and job resources may lead to higher motivation and positive organizational outcomes (when resources buffer the impact of demands), or to the exhaustion of mental and physical energy (when demands are too high). In this paper, we focused on the need for recovery, as negative outcome, and on the work engagement, as positive outcome.

*Need for recovery.* The individual perception of one’s need to restore the energies spent while working is called need for recovery, and it is proportional to the fatigue cumulated during the workday [36]. Typical examples of need for recovery experiences are difficulty to relax at the end of a working day, lack of concentration during their free time after work, need for free days to rest, and tiredness when starting a new workday [37]. If not restored, the subsequent inability to efficiently manage working tasks may trigger a further depletion of resources and long-term detrimental outcomes, such as burnout, strain, prolonged fatigue and psychological distress [38,39]. A study among teachers demonstrated that emotional dissonance and the need for recovery at the end of the working day share a reciprocal influence, in a negative direction [36]. In this study, in agreement with the health-impairment process of the JD-R model [14], surface acting (i.e., the emotional regulation strategy that fakes the required emotional expressions) produces a significant daily resources depletion at work, which results in higher levels of emotional exhaustion and, in turn, a limited number of resources that employees are able to spend during their leisure time. This result suggests that attempting to cope with excessive job demands may lead employees to perceive a complete shortage of their emotional resources (i.e., emotional exhaustion) and subsequently experience a greater need to recover [36,40,41]. 

*Work engagement.* Work engagement can be defined as “the positive, fulfilling, and work-related state of mind that is characterized by vigor, dedication and absorption” ([42], p. 295). Vigor is associated with high levels of energy and mental resilience while working, willingness to invest effort in work, and persistence also in the face of difficulties. Dedication refers to being strongly involved in one’s work and experiencing a sense of significance, enthusiasm, and challenge. Absorption is characterized by being fully concentrated and happily engrossed in the work. Engaged employees have a sense of energetic and affective connection with their work, and they are more inclined to consider it challenging, rather than stressful and demanding [22]. Various job characteristics and management practices function as key causes of engagement. Using the Job Demands–Resources [14] framework, we can expect work engagement to be promoted by job resources, because these “are assumed to activate a motivational process whereby perceived resources that are instrumental in achieving work goals can also foster employees’ growth, learning, and development; satisfy needs for autonomy and competence; and increase willingness to dedicate one’s efforts and abilities to the work task” ([27], p. 836). The JD-R model does not assume a direct relationship between job demands and work engagement. However, research grounded in this perspective has produced conflicting results. In fact, distinguishing among types of demands leads to the discovery of meaningful relationships between demands and engagement. Perhaps counterintuitively, some job demands (for example, workload) can indeed be overtime positively linked with work engagement through the motivational process [43,44]. Job demands such as high workload, time pressures, and high levels of job responsibility can be considered to be examples of challenge stressors [45]. The fact that challenges tend to be appraised as stressful demands that have the potential to promote mastery, personal growth, or future gains, may explain their connection with work engagement. If job demands and engagement are meaningfully related, the direction of the relationship certainly varies as a function of the nature of the demand in question [27]. Emotionally dissonant demands, however, are unequivocally negatively associated with work engagement [44,46].

### 1.4. Hypotheses

In JD-R model [14], stressors are posited to be more strongly related to fatigue, whereas resources are related to work engagement. Consequently, the incidence of demands (workload, mental load and emotional dissonance) and resources (work–life balance, independence and career opportunities) on the need for recovery and on work engagement was verified.

**Hypothesis** **1** **(H1).** *Job demands are positively related to the need for recovery and negatively related to work engagement among university fixed-term researchers.*

**Hypothesis** **2** **(H2).** *Job resources are negatively related to the need for recovery and positively related to work engagement among university fixed-term researchers.*

In this paper, it is assumed that being forced to pretend to feel emotions that are not actually felt (i.e., emotional dissonance) deeply affects researchers’ stress levels [9,10,31]. Although researchers can already expect to face workload and mental load as job demands, as characteristics intrinsic to academic work, emotional dissonance is more difficult to foresee and therefore to bear. We hypothesized that emotional dissonance is not (only) stressful because of its associations with various organizational stressors [33]. Rather, it is an independent stressor that depletes RTDs’ resources and leads to exhaustion.

**Hypothesis** **3** **(H3).** *Emotional dissonance is able to predict a unique portion of variance of the need for recovery among university fixed-term researchers.*

As mentioned, the two types of RTDs do not differ in terms of work content. What differentiates RTDa from RTDb are career prospects (more certain for RTDb) and tenure (lower for RTDa). We assumed that the contract type would not have a specific role in the relationship between job demands and the need for recovery, given the lack of substantial differences in work and mental loads. Instead, we verified the moderating role of the contract type in the relationship between job resources and work engagement, since different contractual status may generate different psychological and motivational dynamics.

**Hypothesis** **4** **(H4).** *Contract type (RTDa or RTDb) moderates the relationship between job resources and work engagement.*

**Figure 1:**
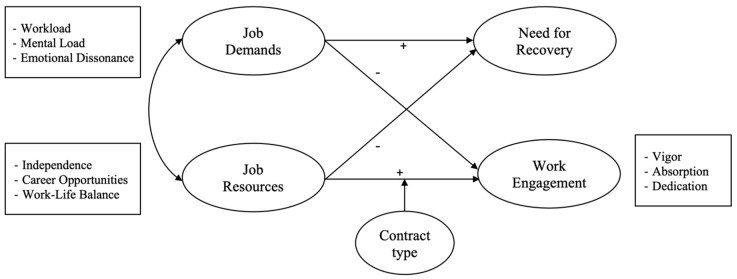
The conceptual model of the hypothesized relationships between variables.

The conceptual model of the hypothesized relationships between variables in shown in Figure 1.

## 2. Materials and Methods 

### 2.1. Sample

The research population for the study consists of university researchers employed on fixed-term contracts at Italian universities. The data were collected through an online questionnaire and the response to all items was mandatory. Prior to data collection, we compiled a list of email addresses of potential participants using the contact information publicly available on the universities’ websites. Potential participants were then contacted by an email that included relevant research information and a direct link to our web-based questionnaire. Of those invited to participate, only 159 completed the questionnaire (approximately 25% of the total emails sent). Two subjects were excluded from the analysis because their working status was no longer fixed-term.

The sample consisted of 157 RTDs (87 males, 70 females), 42% of “type a” (*n* = 66) and 58% of “type b” (*n* = 91), with a mean age of 39 years (*sd* = 5.7). Respondents came from different universities of the territory: 19% from islands (Sicily and Sardinia), 21% from southern Italy, 20% from the center, 23% from north-east and 17% from north-west. 66% of respondents were assigned from 0 to 100 h of lessons per year, 31.5% from 100 to 250, 2.5% more than 250. The 27.7% of the sample has worked as an RTD for less than 1 year, 26.4% for 1 to 2 years, 29.6% for 3 to 5 years, and 16.4% for more than 5 years. In terms of disciplines, 28% of RTDs in our sample worked in technical sciences, 41.3% in natural and medical sciences, 10.9% in economic and juridical sciences, and 19.8% in human and social sciences. The sample is well balanced in terms of gender and type of contract, and well distributed in terms of disciplines, territory, and years of tenure.

### 2.2. Measures

*Work engagement* was measured using the Italian version of the 9-item version of the Utrecht Work Engagement Scale [47], which investigates the experience of three aspects of the construct by three-item scales: *Vigor* (Cronbach’s alpha in our sample = 0.84), *Dedication* (Cronbach’s alpha = 0.82) and *Absorption* (Cronbach’s alpha = 0.69). Responses to items are given on a 6-point Likert scale from (1) “never” to (6) “always”. The sum of the scores was calculated, the range goes from a minimum of 3 to a maximum of 18 for each subscale. 

The Italian version of the Questionnaire on the Experience and Evaluation of Work (QEEW) [48] was used to investigate job demands (workload and mental load), job resources (independence and career opportunities), and fatigue (need for recovery) respectively through the scales *Pace and amount of work*, *Mental load*, *Independence in your work*, *Career possibilities*, and *Need for recovery*. All items of QEEW scales were assessed using a 4-point Likert scale from (1) “never/absolutely no” to (4) “always/absolutely yes”; a higher score indicated a higher presence of the construct. 

*Pace and amount of work* scale (4 items, sum score ranges from 4 to 16) assesses the perceived workload and time pressure. Examples of the items include: “Do you have to work extra hard in order to complete something?”, and “Do you work under time pressure?”. Cronbach’s alpha was 0.70. 

*Mental load* scale (4 items, sum score ranges from 4 to 16) assesses the degree of concentration and attention required to do the job. In our sample it showed a Cronbach’s alpha of 0.70. An example of item is: “Does your work demand a lot of concentration?”. 

*Independence* scale (5 items, sum score ranges from 5 to 20) assesses the worker’s perception about being able to operate independently in the workplace. Examples of the items include: “Do you have influence in the planning of your work activities?”, and “Can you personally decide how much time you need for a specific activity?”. Cronbach’s alpha was 0.85. 

*Career possibilities* scale (3 items, sum score ranges from 3 to 12) assesses the perception of the possibilities offered by the organization to improve employees’ career status. An example of item is: “Does your job give you the opportunity to be promoted?”. Cronbach’s alpha value was 0.75.

*Need for recovery* scale (6 items, sum score ranges from 6 to 24) assesses the feeling of physical and mental fatigue considered excessive and the consequent need for time and space to detach. Examples of the items include: “I find it difficult to relax at the end of a working day”, and “Because of my job, at the end of the working day I feel rather exhausted”. Cronbach’s alpha value was 0.86.

*Emotional Dissonance* (3 items, sum score ranges from 3 to 12) was measured using the *Surface Acting* scale of the Emotional Labor Scale [29], which investigates the discrepancy between the experienced emotions and those required by the organization. For convenience, the affirmative items of the original scale were transformed into questions. All items were assessed using a 4-point Likert scale from (1) “never/absolutely no” to (4) “always/absolutely yes”; a higher score indicated a higher presence of the construct. An example of item is: “In certain circumstances of your work do you have to hide your true feelings?”. Cronbach’s alpha was 0.89. 

Finally, a *Work–Life Balance* scale was created ad hoc to investigate the level of perceived balance between working life and private life. It included 3 items, assessed using a 4-point Likert scale from (1) “never/absolutely no” to (4) “always/absolutely yes”; a higher score indicated a higher presence of the construct. The sum score ranges from 3 to 12. An example of item is: “My organization allows me to carve out time for my hobbies”. Cronbach’s alpha was 0.67.

The questionnaire also included information concerning demographic characteristics of the respondents (age, gender, geographical location), and employment variables (formal position, type of contract, length of employment, hours of lessons assigned).

### 2.3. Data Analysis

Data analysis was performed using SPSS 26 (IBM, Armonk, NY, USA) and Mplus 8 (Mplus, Los Angeles, CA, USA). To test hypotheses 1 and 2, we used the structural equation modeling (SEM) technique. The following criteria were employed to evaluate the goodness of fit: χ^2^ likelihood ratio statistic, Tucker–Lewis index (TLI), comparative fit index (CFI), the root mean square error of approximation (RMSEA) with associated confidence intervals, and the standardized root mean square residual (SRMR). We accepted TLI and CFI values around or greater than 0.90, RMSEA and SRMR values lower than 0.08 [49]. 

Following some results that emerged from the analysis, we carried out a parallel multiple mediation analysis through the Macro PROCESS (version 3.5, by Andrew F. Hayes) of SPSS, in order to assess the contribution of potential mediators of the relationship between mental load and need for recovery. A significant indirect pathway is inferred if the lower and upper limit confidence intervals do not cross zero. In our model mental load was the predictor, the need for recovery was the outcome, emotional dissonance and workload were the parallel multiple mediators. The model ran 5000 bootstrap samples, and 95% confidence intervals are reported.

A multiple hierarchical regression was used to evaluate the hypothesis that emotional dissonance explains additional variance compared to the classic stressors (H3). In the first step, control variables such as gender, years of tenure, and type of RTD contract were introduced. The choice of these variables was suggested by the literature [50], reporting higher levels of emotional labor among women and untenured faculty members. In our sample low tenure means from 0 to 2 years spent on the role (54% of the total sample), and high tenure means from 3 years onwards (46% of the total sample). In the second step, the usual job demands variables were introduced. Finally, in the third step, the variable Emotional Dissonance was introduced, in order to evaluate its unique predictability. If emotional dissonance explains a significant amount of variance in need for recovery, after controlling for workload and mental load, this will imply a unique contribution of emotional stressor to the prediction of fatigue. To examine the moderating effect (H4), another multiple hierarchical regression was used. We included all the controlled variables into the first step, and we added job resources as independent variables in the second step. Finally, we added the interaction terms in the third step. The interaction term was computed by calculating the multiplication of the standardized moderators.

## 3. Results

Correlations between variables, with their means and standard deviations, are shown in Table 1. 

To test Hypotheses 1 and 2, we used the SEM technique. We assessed the impact of demands (workload, mental load and emotional dissonance) and resources (independence, career opportunities and work–life balance) on the need for recovery and on work engagement. The hypothesized model yielded a very good fit: χ^2^ (df = 3) = 3.193, *p* > 0.05; RMSEA = 0.020 (0.000–0.137); CFI = 0.99; TLI = 0.99; SRMR = 0.012. It explained 58% of the variance in Need for Recovery, 45% in Vigor, 36% in Dedication and 21% in Absorption. We expected that all of the job demands variables positively influenced the need for recovery and were negatively related to work engagement dimensions, while all of the job resources variables positively influenced work engagement and were negatively related to need for recovery. The results revealed differences from the initial hypothesis. Some dimensions of work engagement, for example, did not report significant influences from some variables examined, and the same for the need for recovery. Dedication is positively influenced by Independence (β = 0.208 **, se = 0.08), Career Opportunities (β = 0.192 **, se = 0.07), Mental Load (β = 0.344 **, se = 0.07) and negatively by Emotional Dissonance (β = −0.219 **, se = 0.08). No significant effect was found for the Workload variable. Vigor is positively influenced by Independence (β = 0.284 **, se = 0.07), Career Opportunities (β = 0.272 **, se = 0.06) and negatively by Emotional Dissonance (β = −0.282 **, se = 0.08). No significant effect was found for Workload and Mental Load variables. Absorption is influenced only by Mental Load (β = 0.438 **, se = 0.08), probably due to the strong similarity of the two constructs. No significant effect was found for the other variables. The fact that none of the variables identified as job resources has shown to predict Absorption underlines the controversial aspect of this work engagement dimension. Absorption may include an overlapping feature with working excessively and compulsory [51], a tendency that has very little in common with the positive aspects of work and very much in common with the mental overload. Work–Life Balance, although undoubtedly a resource, did not show any predictive value on the work engagement dimensions.

As regards the stress index variable, the Need for Recovery was positively influenced by Workload (β = 0.204 **, se = 0.07) and Emotional Dissonance (β = 0.409 **, se = 0.07), and negatively by Independence (β = −0.159 **, se = 0.07) and Work–Life Balance (β = −0.164 **, se = 0.06). No effect was found for Career Opportunities and Mental Load variables. Therefore, H1 and H2 were only partially confirmed.

**Figure 2:**
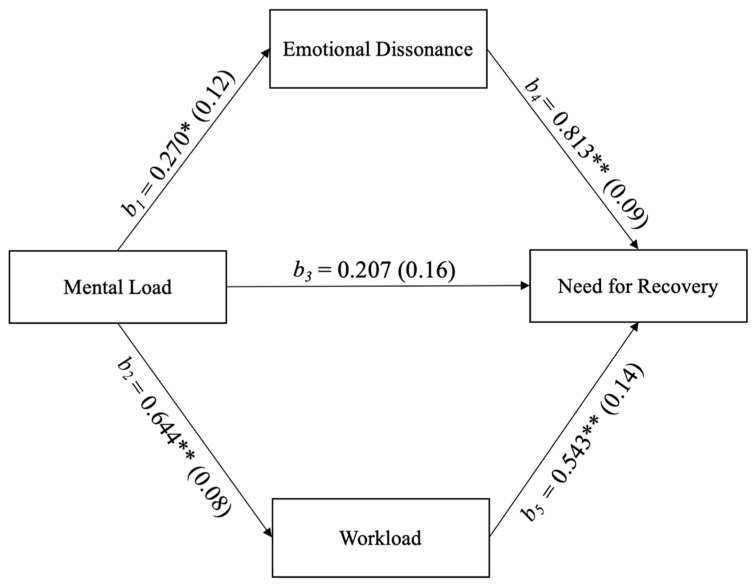
Parallel multiple mediation analysis of the mediators (Emotional Dissonance and Workload) in the relationship between Mental Load and Need for Recovery (*n* = 157). Note: * *p* < 0.05, ** *p* < 0.01.

Mental load, despite its stressor role, did not result in predicting fatigue in our sample. This result has opened to further investigations in order to understand if, rather than directly, the mental load could act on the need for recovery indirectly, through the mediation of workload and emotional dissonance. We therefore carried out a parallel multiple mediation analysis, using the PROCESS Macro (version 3.5) in SPSS. The results are graphically represented in Figure 2.

Results of the mediation analysis show a significant effect of Mental Load on both Emotional Dissonance (b_1_ = 0.270, se = 0.12) and Workload (b_2_ = 0.644, se = 0.08). Overall, the three predictors explain 53% of the variability observed in the Need for Recovery (F (3, 153) = 57.14, *p* = 0.000). Mental Load does not predict Need for Recovery directly (b_3_ = 0.207, se = 0.16, *p* = 0.207, bootstrap 95% C.I. = −0.1159–0.5308), but only indirectly through the effect of Workload and Emotional Dissonance. Both indirect effects, the one of the Mental Load on the Need for Recovery through Emotional Dissonance (b_4_ = 0.813, bootstrap se = 0.09 bootstrap 95% C.I. = 0.0180–0.4433) and the one of the Mental Load on the Need for Recovery through the Workload (b_5_ = 0.543, bootstrap se = 0.14 bootstrap 95% C.I. = 0.1537–0.5566) are significant. In conclusion, the parallel multiple mediation model showed that there is not a significant direct relationship between mental load and need for recovery (i.e., the stress outcome), but significant indirect relationships from mental load to need for recovery through emotional dissonance and workload (i.e., two relevant job demands) was found.

Results from the multiple hierarchical regression analysis used to verify H3 are shown in Table 2. In the first step, control variables such as gender, years of tenure and type of RTD contract were introduced. In the second step, the Workload and Mental Load variables were introduced. Finally, in the third step, the variable Emotional Dissonance was introduced. 

If emotional dissonance explains a significant amount of variance in need for recovery, after controlling for workload and mental load, this will imply a unique contribution of emotional stressor to the prediction of fatigue. Results (Table 2) show that Workload and Mental Load accounted for 34% of variance of Need for Recovery. When Emotional Dissonance entered the regression, it accounted for an additional 22% of the variance. Hypothesis 3 was confirmed. Hierarchical regression analysis also shows that need for recovery has a greater weight for the sample with fewer years spent in the role.

Prior to hypothesis 4 testing, we split the general sample in two groups (RTDa and RTDb) and compared their means with a t-test. A statistically significant difference emerged in the following variables: Workload (RTDa mean = 12.76; RTDb mean = 12.08), Career Opportunities (RTDa mean = 7.14; RTDb mean = 8.25) and Emotional Dissonance (RTDa mean = 7.94; RTDb mean = 6.91). This suggests that RTDa perceive greater workload, greater emotional dissonance but, at the same time, fewer career opportunities, which could be a risk factor for their well-being.

**Figure 3:**
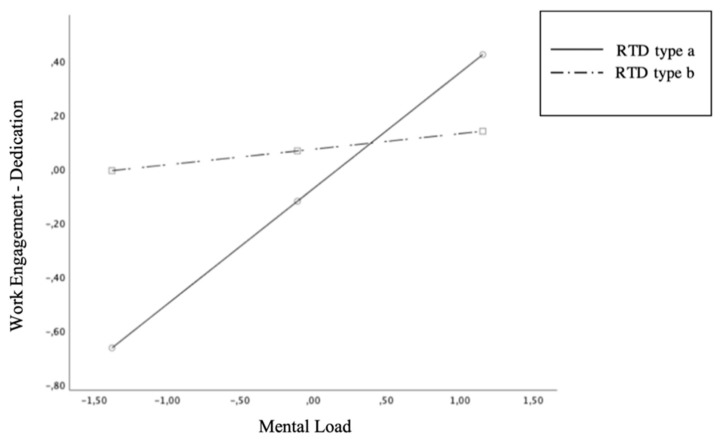
Interaction effect between Mental Load and RTD type (a/b) on Dedication.

Having verified the existence of differences between the two samples, moderation hypotheses (H4) were tested. We investigated whether the interaction between the RTD type of contract and the resources variables could have effects on the work engagement dimensions. Given the absence of significant relationships of the work–life balance variable, this was excluded from the analysis. Furthermore, given the significant path that links mental load to work engagement, we considered Mental Load as a job resource, rather than a job demand. We therefore assessed the interactions of the RTD type of contract with Independence, Career Opportunities, and Mental Load. In the first step, the control variables (gender, years of tenure and type of RTD contract) were introduced. We added job resources as independent variables in the second step. Finally, we added the interaction terms in the third step. As shown in Table 3, the only statistically significant interaction was found in the relationship between Mental Load and Dedication, which showed higher values in the RTDa working situation. H4 was only partially confirmed. The moderation is shown graphically in Figure 3. 

RTDa experiences more dedication than their RTDb colleagues, especially in the situation of high mental load. Since dedication refers to being strongly involved in one’s work and experiencing a sense of significance, enthusiasm, and challenge, this result may be consistent with the feelings experienced by those (the RTDa) who do not have a contract that guarantees linear access to stable academic positions, and whose career progress depends precisely on their enthusiasm and motivation to give their best in the short time that their fixed-term contract grants.

## 4. Discussion

The aim of this research was to focus on fixed-term researchers from Italian universities, a category of temporary workers largely overlooked by the recent literature on work-related stress in academic contexts. We investigated the relationships between some job demands (workload, mental load and emotional dissonance) and some job resources (independence, career opportunities and work–life balance) with, respectively, the need for recovery as negative outcome and the work engagement as positive outcome. In line with the JD-R model [14], we assumed that job demands predicted the need for recovery (i.e., the sense of fatigue experienced at the end of the working day and the consequent need to relax) and that job resources predicted work engagement. As it is a sample of university researchers, whose careers revolve around research, we also assumed that emotional dissonance affected the levels of fatigue and need to recovery more than any other job stressor. Finally, considering the fixed-term nature of the employment contracts of the sample, we wondered whether the contractual typology (type “a” or type “b” RTD) moderated the relationships between job resources and work engagement. 

The results of the analysis confirmed that higher workloads and greater emotional dissonance experienced lead to higher levels of fatigue and need to recover physical and mental energies at the end of the working day. Emotional dissonance, alone, explains the 22% of variance of need for recovery, showing a greater weight than any other job demands on fatigue levels. To better understand this, we need to reflect on the working situation of our sample. In Italy one becomes an RTD after years of PhD. spent studying and doing research. Not all doctoral students deal with teaching and it is indeed very likely that this period distances them from the daily interaction with others, which instead characterizes many other jobs. Perhaps the lack of habit towards teaching increases the discomfort that these researchers experience when, once they become RTDs, they find themselves having to combine research activities with teaching hours. Another explanation for the special weight of emotional dissonance as a stressor among fixed-term researchers might be the lack of autonomy and decision-making power compared to associate professors. In universities, relationships are often asymmetrical and tenured professors usually have more power and autonomy in deciding how to deal with interlocutors of lower power (students, trainees, doctoral students). On the other hand, untenured faculty members are more likely to engage in some form of emotion modification and be much more aware of their emotional displays, since they depend on positive evaluations to launch their academic career on an upward trajectory [50]. As if the high workloads, precariousness, and the pressure of having to publish more and more research articles were not enough, RTDs must also worry about faking emotions that they do not actually feel as an integral part of their work. This seems to contribute more than anything else to the increase in fatigue levels experienced during working hours. In addition, it contributes to decrease the levels of work engagement as well.

On the other hand, a variable initially included among the job demands was found to predict not the negative outcome, but the work engagement instead. The mental load among Italian RTDs predicts dedication and absorption towards their job, while it acts on the need for recovery not directly but indirectly, through workload and emotional dissonance. Despite reporting high levels of stress and job demands, this is the evidence that fixed-term university researchers may also gain a considerable degree of satisfaction from their work, even if precarious and increasingly demanding. It would seem that mental load is a challenging demand, which therefore acts as motivational factor. In fact, even if job resources are expected to be the main source of work engagement, challenging job demands could also trigger positive emotions and positively affect employees’ engagement [29,45]. Employees tend to perceive these demands as opportunities to learn, achieve, and demonstrate the type of competence that tends to get rewarded, therefore they produce engagement just as with any other job resource. In the case of researchers, their mental load is a direct consequence of their research work, of the commitment and concentration that it requires. It is probably the reason they chose to undertake this career path in the first place. Therefore, perhaps it is not seen as a burden that worsens their working days, but as a positive and motivating factor that pushes them to continue to commit and to persist on their path, although precarious.

In line with the JD-R model [14], we then hypothesized that job resources predicted work engagement. The analysis showed a positive influence of career opportunities and independence on vigor and dedication. As hypothesized, greater control over one’s work in terms of task planning and time organization leads to greater vigor in completing one’s work and greater dedication towards it. Likewise, the career opportunities offered by the universities contribute to increasing researchers’ work engagement. Only work–life balance has shown no effect on work engagement among Italian fixed-term researchers, although it significantly reduced the need for recovery levels and negatively correlated with all the job demands. 

The last research hypothesis concerned the moderating effect of the contractual typology (type “a” or type “b” RTD) in the relationship between job resources and work engagement. In light of the results described so far, we have treated the mental load as a job resource, and we have left the work–life balance out of the analysis. The only statistically significant interaction was found in the relationship between mental load and dedication, which showed higher values in the RTDa working situation: researchers in the most precarious condition are likely to be more dedicated to their profession, especially in situations of high mental loads. This discovery opens up many considerations. For example, it could be explained by the different working moment that the two types of researchers are experiencing. The RTDa are still at the beginning of their professional career, therefore they may need a greater motivational drive to continue to engage and not be discouraged by the uncertainties of the future. In fact, the mental load was found to be significantly related to sustained attention (absorption) and sense of purpose (dedication) and therefore is a motivating feature of the researchers’ work. Evidently the greater uncertainty of their working situation, compared to that of their colleagues, does not completely discourage them. Probably a condition of high mental load increases the dedication of the RTDa for the same reason that other challenge factors increase the engagement: because they are experienced as opportunities to learn, achieve, and demonstrate the type of competence that tends to get rewarded [45]. In addition, in fact, the RTDa might have a greater need to demonstrate their skills, since they still have to gain a place within the academy. Furthermore, being at the beginning of one’s career as researchers could bring with it much more enthusiasm and desire to get involved. For the same reason, low mental loads are not experienced well, perhaps because they go hand in hand with little research commitments. The RTDb, however, shows much more dedication in conditions of low mental load than RTDa, and less dedication in conditions of high mental load compared to the colleagues, perhaps because they are already in a more advanced working situation and very little is missing to achieve the goal of becoming associate professors. Therefore, their job security is higher, and their dedication does not show changes related to mental loads because over time it has presumably reached an adequate stability. 

### Limitations and Future Research

The current study has several limitations. The first limitation stems from its cross-sectional nature, meaning that no reliable conclusions can be drawn regarding the causal direction of effects. The second limitation concerns the low response rates. The small size of the sample did not enable us to test more complex statistical models. Another limitation stems from the employing of self-report data only. The need for recovery and the work engagement could also be explained by other variables not included in the study. This highlights the synergistic nature of influences affecting the workplace environment and the difficulties faced by researchers in searching out the most influential.

The present study can be improved by taking into consideration several other variables and combined measures, for example, by associating self-report with the fatigue-inducing mental tasks and consequent measurements of the physiological responses with EEG, or by evaluating other indices, such as heartbeat or sleep quality. A longitudinal research design should be preferred in order to better evaluate the influence of job insecurity on stress levels. In this study, we only use the type of contract (assuming that type a RTDs have a higher level of job insecurity than type b RTDs) to verify any differences between the two groups of researchers in the relationships between job resources and work engagement. Future research developments, however, may consider a more structured construct of job insecurity to investigate further. To shed further light on the differences that emerged between RTDa and RTDb in terms of dedication and mental load, it would also be interesting to compare the responses obtained by fixed-term researchers with those of tenured professors.

## 5. Conclusions

Altogether, the present study provided new insights into the relationship between job insecurity, demands and engagement by showing that even a precarious type of contract can boost dedication in conditions of high mental loads. Even though it does not change their insecure job situation, mental load can act positively on the well-being of researchers, stimulating their engagement and motivating them to pursue their career goals. However, we also demonstrated the weight of emotional dissonance in the academic context. We proved that emotional dissonance is not (only) stressful because of its associations with other job stressors. Rather, being forced to fake emotions that are not actually felt deeply affects researchers’ stress levels in its own way, acting as an independent source of exhaustion that must be addressed to protect the well-being of researchers, even if only for a fixed term.

## Figures and Tables

**Table 1:** Means, standard deviations, and correlations between study variables (*n* = 157).

Variable	Mean (*sd*)	1	2	3	4	5	6	7	8	9	10
1. Workload	12.36 (2)	1									
2. Mental Load	14.17 (1.6)	0.509 **	1								
3. Emotional Dissonance	7.34 (2.5)	0.482 **	0.166 *	1							
4. Independence	15.13 (2.7)	−0.369 **	−0.062	−0.530 **	1						
5. Career Opportunities	7.78 (2.2)	−0.167 *	0.022	−0.322 **	0.370 **	1					
6. Work-Life Balance	7.55 (1.9)	−0.433 **	−0.248 **	−0.474 **	0.443 **	0.262 **	1				
7. Need for Recovery	14.9 (4)	0.566 **	0.308 **	0.668 **	−0.510 **	−0.231 **	−0.526 **	1			
8. Work Eng—Vigor	11.75 (3.3)	−0.170 *	0.101	−0.506 **	0.525 **	0.476 **	0.327 **	−0.411 **	1		
9. Work Eng—Absorption	13.13 (2.4)	0.0233 **	0.461 **	0.053	−0.035	0.083	−0.106	0.137	0.377 **	1	
10. Work Eng—Dedication	13.74 (2.9)	−0.155	0.232 **	−0.385 **	0.412 **	0.368 **	0.235 **	−0.307 **	0.807 **	0.480 **	1

Note: * Correlation is significant at the 0.05 level (2-tailed). ** Correlation is significant at the 0.01 level (2-tailed).

**Table 2:** Coefficients table of hierarchical regression analysis with Need for Recovery as outcome (*n* = 157).

Predictors	Need for Recovery			
	R^2^	ΔR^2^	β	*p*
*Step 1—Control variables*	0.03			
Gender (−0.5 = male; 0.5 = female)			0.051	0.354
Tenure (−0.5 = low; 0.5 = high)			**−0.183**	**0.002**
RTD type (−0.5 = a; 0.5 = b)			0.088	0.135
*Step 2—Job Demands*	0.34	0.31		
Workload			**0.305**	**0.000**
Mental load			0.062	0.334
*Step 3*	0.56	0.22		
Emotional dissonance			**0.562**	**0.000**

Note: β = standardized beta-coefficient from the final step. Significant regression coefficients are shown in boldface.

**Table 3:** Coefficients table of hierarchical regression analysis with Work Engagement dimensions as outcomes (*n* = 157).

Predictors	Vigor				Absorption				Dedication			
	R^2^	ΔR^2^	β	*p*	R^2^	ΔR^2^	β	*p*	R^2^	ΔR^2^	β	*p*
*Step 1—Control variables*	0.05				0.00				0.03			
Gender (−0.5 = male; 0.5 = female)			−0.037	0.574			0.006	0.932			−0.006	0.935
Tenure (−0.5 = low; 0.5 = high)			−0.079	0.238			0.015	0.840			−0.090	0.203
RTD type (−0.5 = a; 0.5 = b)			0.020	0.774			−0.016	0.841			−0.021	0.772
*Step 2—Job Resources*	0.39	0.34			0.22	0.22			0.30	0.27		
Independence			**0.434**	**0.000**			−0.018	0.827			**0.360**	**0.000**
Career opportunities			**0.295**	**0.000**			0.078	0.338			**0.224**	**0.004**
Mental load			**0.129**	**0.047**			**0.462**	**0.000**			**0.269**	**0.000**
*Step 3—Interactions*	0.41	0.02			0.24	0.02			0.33	0.03		
Independence X RTD type			−0.125	0.079			−0.102	0.206			−0.080	0.287
Career opportunities X RTD type			0.013	0.848			−0.009	0.914			0.034	0.645
Mental load X RTD type			−0.070	0.186			−0.087	0.243			**−0.171**	**0.015**

Note: β = standardized beta-coefficient from the final step. Significant regression coefficients are shown in boldface.

## Data Availability

The data presented in this study are available on request from the corresponding author. The data are not publicly available due to privacy reasons.
